# Time-dependent changes in pulmonary surfactant function and composition in acute respiratory distress syndrome due to pneumonia or aspiration

**DOI:** 10.1186/1465-9921-8-55

**Published:** 2007-07-27

**Authors:** Reinhold Schmidt, Philipp Markart, Clemens Ruppert, Malgorzata Wygrecka, Tim Kuchenbuch, Dieter Walmrath, Werner Seeger, Andreas Guenther

**Affiliations:** 1 University of Giessen Lung Center (UGLC), Medical Clinic II, Giessen, Germany

## Abstract

**Background:**

Alterations to pulmonary surfactant composition have been encountered in the Acute Respiratory Distress Syndrome (ARDS). However, only few data are available regarding the time-course and duration of surfactant changes in ARDS patients, although this information may largely influence the optimum design of clinical trials addressing surfactant replacement therapy. We therefore examined the time-course of surfactant changes in 15 patients with direct ARDS (pneumonia, aspiration) over the first 8 days after onset of mechanical ventilation.

**Methods:**

Three consecutive bronchoalveolar lavages (BAL) were performed shortly after intubation (T0), and four days (T1) and eight days (T2) after intubation. Fifteen healthy volunteers served as controls. Phospholipid-to-protein ratio in BAL fluids, phospholipid class profiles, phosphatidylcholine (PC) molecular species, surfactant proteins (SP)-A, -B, -C, -D, and relative content and surface tension properties of large surfactant aggregates (LA) were assessed.

**Results:**

At T0, a severe and highly significant reduction in SP-A, SP-B and SP-C, the LA fraction, PC and phosphatidylglycerol (PG) percentages, and dipalmitoylation of PC (DPPC) was encountered. Surface activity of the LA fraction was greatly impaired. Over time, significant improvements were encountered especially in view of LA content, DPPC, PG and SP-A, but minimum surface tension of LA was not fully restored (15 mN/m at T2). A highly significant correlation was observed between PaO_2_/FiO_2 _and minimum surface tension (r = -0.83; p < 0.001), SP-C (r = 0.64; p < 0.001), and DPPC (r = 0.59; p = 0.003). Outcome analysis revealed that non-survivors had even more unfavourable surfactant properties as compared to survivors.

**Conclusion:**

We concluded that a profound impairment of pulmonary surfactant composition and function occurs in the very early stage of the disease and only gradually resolves over time. These observations may explain why former surfactant replacement studies with a short treatment duration failed to improve outcome and may help to establish optimal composition and duration of surfactant administration in future surfactant replacement studies in acute lung injury.

## Background

Pulmonary surfactant, which covers the large alveolar surface in all mammalian species investigated, is composed primarily of phospholipids (80–85%), with dipalmitoylated phosphatidylcholine (DPPC) predominating (~50% of all PC species). It also contains neutral lipids (10%) and surfactant-specific proteins (SP-A, SP-B, SP-C, SP-D; together 5–10%) [1,2]. By reducing alveolar surface tension, pulmonary surfactant stabilizes the alveoli and prevents them from collapse. Alterations to the pulmonary surfactant system have long been implicated in the course of inflammatory lung diseases such as the Acute Respiratory Distress Syndrome (ARDS). Indeed, in clinical studies focusing on ARDS [3-6] and, more recently, on severe pneumonia [6], a marked impairment of surface activity of surfactant isolates from BALF has been documented. To date, most attention has been focused on the analysis of the phospholipid profiles and the apoprotein content of surfactant from patients with ARDS. SP-A [5,6], SP-B [5,6] and SP-C levels [7] were decreased, the relative phosphatidylcholine palmitic acid content was reduced [3,8], and a marked reduction in phosphatidylglycerol (PG) has been observed throughout. In addition, the inhibitory action of fibrin(ogen) [9] and other plasma proteins [10] entering the alveolar space, proteases [11], phospholipases [12] and reactive oxygen species [13] on surfactant function has been described.

Despite advances in the field of intensive care medicine, ARDS is still characterized by high mortality rates (30–40%) and the only successful medical intervention that significantly reduces mortality is a protective lung ventilation strategy [14]. Pharmacological interventions, although assessed in numerous clinical studies, have all failed to exert a significant influence on outcome [15]. In view of transbronchial surfactant application, recent studies revealed that it is possible to beneficially affect gas exchange in patients with early ARDS if the appropriate material and dose is applied [16-19]. Pulmonary shunt flow, the predominant gas exchange abnormality in ARDS patients, is largely reduced upon transbronchial application of 300 mg/kg body weight of a natural surfactant preparation (Alveofact^®^) in ARDS patients, alongside with a significant improvement in surface activity of the alveolar surfactant pool [20,21]. Similarly, improvement of gas exchange has been encountered in two large phase III studies assessing the efficacy of a recombinant SP-C based surfactant preparation (Venticute^®^) in early ARDS subjects [17,22]. In these patients, surfactant was administered up to four times within a treatment window of 24 h. Despite the beneficial effect on gas exchange throughout this treatment window, duration of mechanical ventilation and outcome remained unaffected by Venticute^® ^treatment. Two possible explanations exist for the observed failure of Venticute^® ^treatment to improve outcome in these patients: i) the profound impact of non-pulmonary organ failure on outcome in indirect forms of ARDS (pancreatitis, trauma, non-pulmonary sepsis) and ii) the potentially short duration of treatment (first 24 h after inclusion).

Indeed, robust data on the time-course of surfactant changes in acute inflammatory lung diseases are limited, either due to the time period investigated in observational studies, or to the restricted number of parameters analyzed [4,23,24]. However, data regarding the time-course and duration of surfactant alterations in ARDS patients may help to understand why surfactant replacement studies with a short treatment duration failed to improve outcome and may help to determine the optimal timing and duration of exogenous surfactant administration and the optimal composition of the exogenous surfactant material. We, therefore, analyzed biochemical and biophysical surfactant properties in 15 patients with direct ARDS at three different time points over an observation period of 8 days after onset of mechanical ventilation (< 24 h, ~4 days and ~8 days).

## Methods

### Patient Population

Patients were recruited at the intensive care unit of the Department of Internal Medicine of the Justus-Liebig-University in Giessen, Germany between 1999 and 2002. The study protocol was approved by the local ethics committee, and informed consent was obtained from either the patient or next of kin.

15 German patients with direct ARDS due to pneumonia (n = 13) or aspiration (n = 2) were included. All patients are of caucasian origin. The inclusion criteria included: age between 18 and 70 years; diagnosis of ARDS according to the Consensus Conference Criteria [25] due to aspiration (if witnessed) or pneumonia (if one major (cough, sputum production, fever) and two minor (dyspnea, pleuritic chest pain, altered mental status, pulmonary consolidation by physical examination, total leukocyte count > 12000/mm^3^) criteria were fulfilled) [26].

Exclusion criteria included the following: pregnancy, acute myocardial infarction, left heart failure (pulmonary capillary wedge pressure > 18 mm Hg as assessed by a pulmonary-artery catheter or missing evidence in echocardiography), lung contusion, any preexisting lung disease (e.g. fibrosis, chronic obstructive lung disease) with a FEV_1 _or FVC ≤ 65% predicted, malignant underlying disease including primary cancer of the lung or cancer metastatic to the lung, immunosuppressive drugs and leukopenia (white blood cells < 1000/μl), severe traumatic or hypoxic brain injury, additional investigational drugs.

All patients required mechanical ventilation. Respirator settings were chosen according to the individual requirements. General therapeutic approaches included intravenous volume substitution, low-dose heparin application, parenteral nutrition, antibiotic drug therapy, and administration of vasoactive or inotropic drugs, when indicated. The main demographic and clinical data of the patient group are summarized in Table 1.

**Table 1:** Clinical and basic BALF data and cell counts^§^

	**ARDS**			**Control**
	**T0 (<24 h)**	**T1 (3–5 d)**	**T2 (7–9 d)**	

**Time point of BAL**	12.1 ± 1.3 hours	3.9 ± 0.6 days	8.1 ± 1.0 days	
**Number of subjects**	15	15	15	15
**Age [years]**	52.1 ± 3.7 ***			30.8 ± 3.2
**Sex f/m**	7/8			9/6
**Current smoker [n]**	1			
**Ex smoker [n]**	5			
**Never smoker [n]**	9			
**Ethnic origin:**				
**Caucasian**	15			
**PaO_2_/FiO_2 _[mm Hg]**	127.8 ± 10.5 ***	129.1 ± 9.9	200.7 ± 24.1 §	511.1 ± 23.2
**PEEP [cm H_2_O]**	8.4 ± 0.8	8.4 ± 0.7	7.4 ± 0.7	
**Tidal Volume [ml/kg bw]**	9.4 ± 0.8	9.0 ± 0.6	8.9 ± 0.6	
**PIP [cm H_2_O]**	24.9 ± 1.3	25.8 ± 1.5	26.3 ± 1.9	
**APACHE II score**	19.2 ± 2.2	21.5 ± 2.0	19.3 ± 1.8	
**Patients alive day 28 [n]/%**	9/60%			
**Time of ventilation [d]**	40.9 ± 10.8			
**Ventilator-free days at day 28 [d]**	1.6 ± 1.4			
				
**Recovery BALF [%]**	61.1 ± 3.6 ***	55.9 ± 4.6	65.1 ± 4.6	82.0 ± 2.0
**Neutrophils [%]**	60.8 ± 6.5 ***	47.5 ± 8.8 §	23.0 ± 7.3 §§§	0.8 ± 0.2
**Lymphocytes [%]**	7.2 ± 5.1	7.7 ± 4.3	7.6 ± 3.1	4.4 ± 0.8
**Macrophages [%]**	32.0 ± 5.2 ***	44.8 ± 8.1 §	69.4 ± 7.1 §§§	94.8 ± 0.8
**Total protein [μg/ml]**	1139 ± 240 ***	886 ± 167	384 ± 85 §§§	76 ± 8

^§ ^All data are given as mean ± standard error. PaO_2_/FiO_2 _= mean oxygen tension in arterial blood/inspiratory oxygen fraction. PEEP = positive end-expiratory pressure. PIP = peak inspiratory pressure. APACHE II = scores on the acute physiology and chronic health evaluation. Cell counts and total proteins were measured in BALF. Tidal volume is expressed per ideal body weight. Control = healthy volunteers; ARDS = Acute respiratory distress syndrome. *** = p < 0.001: T0 compared to healthy controls (Mann-Whitney-U test) and § = p < 0.05; §§§ = p < 0.001: T1, T2 compared to T0 (Wilcoxon test).

The control group consisted of 15 spontaneously breathing healthy German volunteers, all never smokers, with normal pulmonary function and without any history of cardiac or lung disease (medical staff from the Department of Internal Medicine or medical students from the Medical School of the Justus-Liebig University Giessen, Germany). All controls underwent a detailed medical, drug and tobacco history, a physical examination, an electrocardiogram, clinical laboratory tests (hematology, clinical chemistry, coagulation), and pulmonary function prior to inclusion into the study.

### Study design and bronchoscopy

It was predefined that patients would have to undergo three repetitive BALs, the first within 24 h after intubation (T0), the second between four and five days, and the last one between seven and nine days (T2) after intubation. The average time from diagnosis of ARDS to initial BAL was 21 ± 2 hours. Patients that were originally included into the study but dropped out later either due to extubation (n = 2) or death (n = 4) were excluded from data analysis.

Flexible fiberoptic bronchoscopy was performed in patients and controls by one physician in a standardized manner as previously described [6]. The first BAL was performed in the middle lobe or lingua, the second in the respective contralateral segment and the third in the same segment as the first. A lavage volume of 200 ml of sterile normal saline in ten equal aliquots was used. The recovered bronchoalveolar lavage fluid (BALF) was pooled, filtered through sterile gauze, and immediately centrifuged (300 × g, 10 min, 4°C) to remove cells and membraneous debris. The aliquoted supernatant was subsequently frozen and kept at -80°C until further use. Sedimented BALF cells were resuspended in saline solution, counted and subjected to a cyto spin maneuver [27]. Staining was performed according to the Papenheim method (2 min in May-Grünwald solution, followed by 10 min in Giemsa solution and final rinsing with water).

### Lipid and protein analysis

Lipids were extracted from BALF with chloroform/methanol, and phospholipid content was determined by spectrophotometric measurement as previously described [6]. Total proteins were analyzed using a commercial assay (BCA assay, Pierce, Bonn, Germany).

Phospholipid classes were separated by means of high performance thin-layer chromatography (HPTLC), with subsequent selective staining and densitometric scanning as described previously [6]. The profile of molecular species of phosphatidylcholine from large surfactant aggregates was analyzed after phospholipolytic cleavage of the polar headgroup with phospholipase C and conversion of the resulting diradylglyceroles (DRG) to naphthylurethanes by means of high performance liquid chromatography (HPLC) following a variation of the method described by Rüstow et al. [28]. Due to a lack of material, PC molecular species were only calculated in 10 out of 15 patients.

Surfactant proteins were analyzed in large surfactant aggregates (SP-A, SP-B, SP-C) or in BALF (SP-D): Surfactant protein A (SP-A) was measured using an ELISA protocol as originally described [6]. Surfactant protein B (SP-B) was quantified by an ELISA method as described by this group [29] using a monoclonal antibody directed against porcine SP-B with cross reactivity towards human SP-B and human SP-B as standard. Surfactant protein C (SP-C) was determined by means of an ELISA technique recently described [7], using a polyclonal antibody directed against human recombinant SP-C and human recombinant SP-C as standard. SP-D was measured using a sandwich ELISA with two monoclonal antibodies (IE11, VIF11-Biotin, Bachem, Heidelberg, Germany) and human SP-D as standard, as described previously [30].

### Isolation of large surfactant aggregates and surface tension measurements

Frozen aliquots of BALF were thawn and then centrifuged (48 000 × g, 1 h, 4°C), separating large and small surfactant aggregates [5,31]. The pellet was resuspended in a small volume of 0.15 M (m/v) NaCl/3 mM CaCl_2_, and assessed for PL content. The pellets were then adjusted to a concentration of 2 mg/ml PL, vortexed for 1 min, and used for surface tension measurement, which was performed with a pulsating bubble surfactometer (Electronetics, New York, NJ, USA) as previously described [32,9]. The surface tension after 5 min of film oscillation at minimum bubble radius (γ min) and after 11 s film adsorption (γ ads) is given. Due to the limited amount of large surfactant aggregates, complete data sets of surface tension values (T0, T1 and T2) were only measured in six patients.

### Statistical analysis

All data are given as mean ± standard error. Statistical analysis of differences between i) patients and healthy controls and between ii) surviving and non-surviving patients was performed by testing principle significance diversity first (Kruskal-Wallis-H test), followed by comparison with a non-parametric test (Mann-Whitney-U test). Patient values significantly different from control are indicated with: * = p < 0.05, ** = p < 0.01, *** = p < 0.001. Values significantly different between surviving and non-surviving patients are indicated with: # = p < 0.05, ## = p < 0.01, ### = p < 0.001. Statistical analysis of differences between T0 and T1/T2 values were analyzed with Wilcoxon's matched-pairs signed-ranks test. Patient values different from T0 values are indicated with: § = p < 0.05, §§ = p < 0.01, §§§ = p < 0.001.

## Results

### Clinical and basic BALF data

As summarized in Table 1, the patient cohort exhibited severe limitation in gas exchange at the time of the first BAL (T0), with a PaO_2_/FiO_2 _ratio of 127.8 ± 10.5 mm Hg. Values progressively improved during the following eight days, but remained markedly decreased when compared to control values at T2 (Table 1). At the time of the first BAL (T0), patients were ventilated with an average PEEP of 8.4 ± 0.8 cm H_2_O and a PIP of 24.9 ± 1.3 cm H_2_O. The tidal volume was 9.4 ± 0.8 ml/kg bodyweight, and the APACHE II scores ranged at 19.2 ± 2.2 at the time of the first BAL (Table 1). Both the described ventilator settings and the APACHE II scores did not change significantly during the observation period compared to the initial time point. Six of the 15 patients died within 28 days and the average ventilator-free days accounted for 1.6 ± 1.4 days.

The recovery of the BALF was approximately 20% lower in patients compared to controls and did not change during the observation period (Table 1). In the BALF obtained at T0, neutrophils were the predominant cell type in the cell differential. Later in the time-course, alveolar macrophages gradually increased and neutrophils declined (Table 1). At T2, however, the cell differential was yet not normalized compared to controls (Table 1). Similarly, a marked protein load of the alveolar compartment was encountered at T0 that gradually resolved during the following 8 days. However, at T2, protein concentration in BALF remained five-fold elevated, compared to healthy controls (Table 1).

### Early changes in surfactant properties

**Figure 1:**
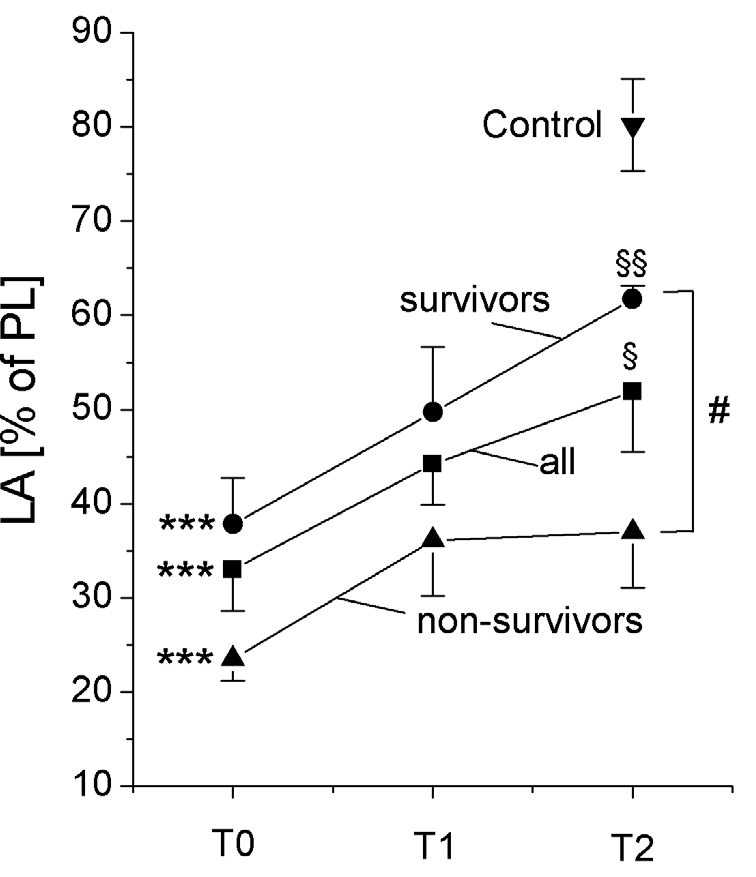
Relative content of large surfactant aggregates (in percent (w/w) in total BALF phospholipids). All data are given as mean ± standard error. *** = p < 0.001: T0 compared to healthy controls (Mann-Whitney-U test); § = p < 0.05; §§ = p < 0.01: T2 compared to T0 (Wilcoxon test); # = p < 0.05: non-survivors compared to survivors (Mann-Whitney-U test).

**Figure 2:**
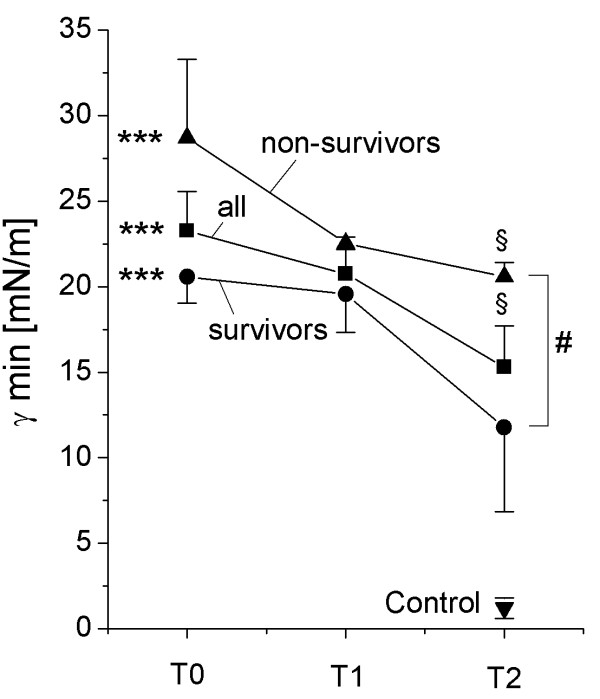
Minimum surface tension. The surface tension of large surfactant aggregates after 5 min film oscillation at minimum bubble radius (γ min) is given. All data are given as mean ± standard error. *** = p < 0.001: T0 compared to healthy controls (Mann-Whitney-U test); § = p < 0.05; §§ = p < 0.01: T2 compared to T0 (Wilcoxon test); # = p < 0.05: non-survivors compared to survivors (Mann-Whitney-U test). Due to the limited amount of large surfactant aggregates, complete data sets of surface tension values (T0, T1 and T2) were only measured in 6 patients (3 survivors, 3 non-survivors).

At T0 and thus ~12 h after intubation, severe and highly significant alterations to the surfactant system were encountered, with a ten-fold reduced phospholipid-to-protein ratio (Table 2), a large reduction in the relative amount of large surfactant aggregates (LA, Figure 1), a pronounced disturbance to the phospholipid and PC molecular species profile (Table 2) and a significant loss of all surfactant proteins (Table 2). In view of the phospholipid and PC molecular species profile, significant reductions in PC and phosphatidylglycerol (PG) were observed with a concomitant increase in the proportion of phosphatidylserine (PS), phosphatidylinositol (PI), phosphatidylethanolamine (PE) and sphingomyelin (SPH). Within the PC fraction of the LA, a dramatic reduction in dipalmitoylated PC species (DPPC), down to less then half of what was measured in healthy controls was observed, and was paralleled by a marked increase in unsaturated species (most of all 16:0/18:1 and 16:0/18:2, Table 2). The hydrophobic surfactant proteins SP-B and SP-C as well as SP-A, but not SP-D, were found to be significantly reduced (Table 2). As a result, the surface tension after 11 sec film adsorption (γ ads) and after 5 min of film oscillation at minimum bubble radius (γ min) was dramatically increased (Table 2, Figure 2).

**Table 2:** Total phospholipids and surfactant apoprotein concentration, phospholipid profiles, phosphatidylcholine molecular species, and adsorption properties of LA^§^

	**ARDS**			**Control**
	**T0 (<24 h)**	**T1 (3–5 d)**	**T2 (7–9 d)**	

**Total Phospholipids [μg/ml]**	31.9 ± 5.0	24.3 ± 4.2	27.9 ± 5.1	35.9 ± 4.8
**Phospholipid-to-protein ratio**	0.043 ± 0.010 ***	0.052 ± 0.021	0.109 ± 0.024 §§	0.461 ± 0.030
				
**PC [%]**	73.3 ± 2.0 **	72.4 ± 3.6	74.7 ± 2.2	81.3 ± 1.6
**PG [%]**	3.6 ± 0.9 ***	2.8 ± 0.8	5.3 ± 0.7 §	11.6 ± 1.1
**PS [%]**	6.5 ± 1.1 ***	5.4 ± 0.9	6.1 ± 1.2	1.8 ± 0.7
**PI [%]**	5.3 ± 0.9 *	5.3 ± 1.2	5.9 ± 1.3	2.5 ± 0.3
**PE [%]**	4.8 ± 1.0 *	6.9 ± 1.6	3.3 ± 0.8	1.4 ± 0.4
**SPH [%]**	5.0 ± 0.7 ***	5.6 ± 0.9	2.9 ± 0.7 §	0.6 ± 0.3
				
**PC 16:0/16:0**	26.81 ± 1.61 ***	32.98 ± 2.36 §	40.16 ± 1.89 §§§	60.65 ± 1.27
**PC 16:0/18:1**	20.43 ± 1.71 ***	15.37 ± 2.06 §	12.57 ± 0.79 §§	9.68 ± 0.49
**PC 16:0/18:2**	10.33 ± 0.80 ***	10.00 ± 1.49	8.53 ± 0.55 §	5.43 ± 0.31
**PC 16:0/16:1**	7.76 ± 0.53 *	6.46 ± 0.40 §	9.79 ± 0.80 §	4.84 ± 0.55
**PC 16:0/14:0**	6.76 ± 1.50	6.39 ± 0.51	7.67 ± 1.44	7.18 ± 0.64
**PC 16:0/20:4**	4.36 ± 0.46 **	2.63 ± 0.32 §	2.30 ± 0.55 §	1.95 ± 0.19
**PC 18:0/18:2**	4.52 ± 0.47 *	3.99 ± 0.39	2.98 ± 0.45 §	1.54 ± 0.45
**PC 18:1/18/1**	4.00 ± 0.23	3.70 ± 0.61	2.52 ± 0.22 §	3.22 ± 0.39
**PC 18:0/18:1**	2.67 ± 0.37	3.66 ± 0.52 §	2.02 ± 0.18	2.14 ± 0.29
				
**SP-A [% PL]**	2.61 ± 0.32 **	3.07 ± 0.57	3.61 ± 0.27 §	4.12 ± 0.09
**SP-B [% PL]**	2.29 ± 0.32 **	2.93 ± 0.43	2.94 ± 0.41	3.71 ± 0.32
**SP-C [% PL]**	1.19 ± 0.25 *	1.34 ± 0.23	1.37 ± 0.26	2.28 ± 0.22
				
**SP-D [ng/ml]**	14.9 ± 2.9	18.8 ± 7.1	18.4 ± 5.8	14.2 ± 2.2
				
**γ ads [mN/m]**	39.0 ± 1.3	33.8 ± 4.7	33.2 ± 3.7	19.1 ± 0.9

^§ ^All data represent mean ± standard error. Control = healthy volunteers; ARDS = acute respiratory distress syndrome. Phospholipid-to-protein ratio and phospholipid profiles were determined in bronchoalveolar lavage fluids (BALF). Each phospholipid class is depicted as percent of all phospholipids. All data are given as mean ± standard error. Definitions of abbreviations: PC = phosphatidylcholine; PG = phosphatidylglycerol; PS = phosphatidylserine; PI = phosphatidylinositol; PE = phosphatidylethanolamine; SPH = sphingomyelin. Values for lysophosphatidylcholine and cardiolipin were not given due to their low content in patients and controls. Phosphatidylcholine molecular species were determined in large surfactant aggregates (LA). Each molecular species is depicted as percent (mol/mol) of all molecular species. Molecular species with a relative content lower than 2% (14:0/14:0, 18:0/18:0, 16:0/22:6, 18:0/20:4, 18:1/18:2, 18:0/14:0) are not given. No significant statistical difference in these molecular species between ARDS at T0 and healthy controls and ARDS at T0 and T1 or T2, respectively, was observed. All data are given as mean ± standard error. Definitions of abbreviations: 14:0 = myristic acid; 16:0 = palmitic acid; 16:1 = palmitoleic acid; 18:0 = stearic acid; 18:1 = oleic acid; 18:2 = linoleic acid; 20:4 = arachidonic acid. Surfactant apoproteins SP-A, SP-B and SP-C were determined in large surfactant aggregates and were depicted as percent (w/w) of phospholipids. Surfactant apoprotein D was determined in bronchoalveolar lavage fluid (BALF) and is given in ng/ml. Surface tension of LA after 11 sec film adsorption (γ ads) is given in mN/m. * = p < 0.05; ** = p < 0.01; *** = p < 0.001: T0 compared to healthy controls (Mann-Whitney-U test) and § = p < 0.05; §§ = p < 0.01; §§§ = p < 0.001: T1, T2 compared to T0 (Wilcoxon test).

### Time course of surfactant changes

In general, a modest improvement in surfactant composition and function was encountered at T1, and – even more evident – at T2. In detail, the relative content of large surfactant aggregates significantly increased at T1 and T2 (Figure 1). The phospholipid profile improved especially in view of phosphatidylglycerol (Table 2) and analysis of the molecular species of PC indicated a clear and highly significant increase in the extent of dipalmitoylation, although the values were clearly below the control range (Table 2). Correspondingly, the relative amount of PC molecular species with unsaturated fatty acids diminished over time (Table 2). The relative amount (compared to PL) of SP-A, SP-B and SP-C in large surfactant aggregates increased and – especially in case of SP-A – normal values were observed at T2. As a result, the surface tension-reducing properties significantly improved over time, although markedly elevated γ min and γ ads values were still observed (Figure 2).

### Differences between surviving and non-surviving patients/Outcome analysis

To investigate a potential role for surfactant measurements as outcome parameter, biochemical and biophysical surfactant characteristics as well as clinical parameter were analysed in surviving (SURV) and non-surviving (non-SURV) patients and significant differences were observed between these groups. Throughout the observation period, APACHE II scores were significantly lower in surviving patients compared to non-surviving patients (T0: SURV 16.3 ± 1.5; non-SURV: 25.8 ± 2.5; p < 0.01; T2: SURV: 17.0 ± 1.2; non-SURV: 24.5 ± 1.9; p < 0.01). Concerning clinical data, the PaO_2_/FiO_2 _was not different at T0, but was significantly different at T2 between survivors (221 ± 17 mmHg) and non-survivors (173 ± 24 mmHg; p < 0.05). PEEP and PIP values were not different between surviving and non-surviving patients. Tidal volumes were significantly higher in non-surviving patients at T0, but not at T1 and T2 (T0: SURV 8.3 ± 0.4 ml/kg bw; non-SURV: 12.3 ± 1.1 ml/kg bw; p < 0.01; T2: SURV 8.4 ± 0.3 ml/kg bw; non-SURV: 9.8 ± 1.3 ml/kg bw). The relative neutrophil counts were not significantly different at T0 and T1, but were significantly lower at T2 in surviving patients (SURV 17.1 ± 3.9; non-SURV: 29.8 ± 4.2; p < 0.05).

The relative content of large surfactant aggregates was significantly lower in non-surviving patients (Figure 1). The relative content of phosphatidylglycerol was lower in non-surviving patients throughout the observation period, however, this decrease was not significant. No significant differences between surviving and non-surviving patients were found in the phosphatidylcholine molecular species profile and in relative content of SP-A, SP-B and SP-D. SP-C levels in large surfactant aggregates at T2 were significantly lower in non-survivors compared to surviving patients (T2: SURV 0.67 ± 0.05% of PL; non-SURV: 0.37 ± 0.06% of PL; p < 0.01). The values for minimum surface tension (γ min) of large surfactant aggregates were significantly lower in surviving patients compared to non-survivors (Figure 2).

### Correlational analysis

**Figure 3:**
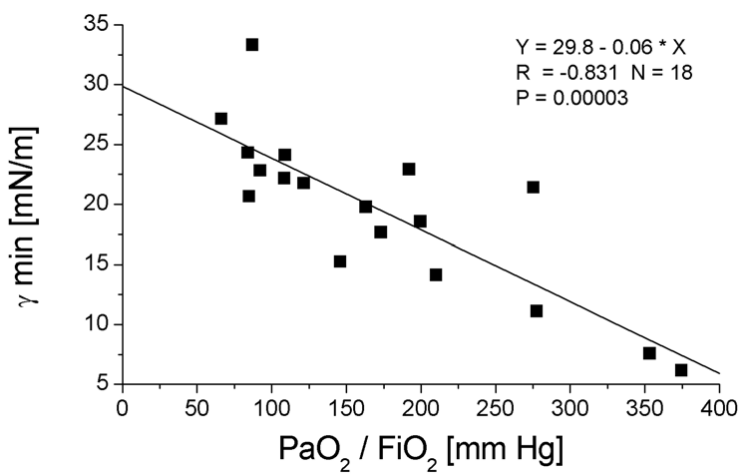
Correlation between the minimum surface tension of the surfactant isolates (γ min) and the PaO_2_/FiO_2 _ratio (mean oxygen tension in arterial blood/inspiratory oxygen fraction) in ARDS patients at T0, T1 and T2. The Pearson correlation coefficient r is given. Due to the limited amount of large surfactant aggregates, complete data sets of surface tension values (T0, T1 and T2) were only measured in six patients.

Pearson correlation was performed between (A) surfactant compositional and functional parameters and PaO_2_/FiO_2_, and (B) between surfactant components and minimum surface tension of the surfactant isolates. Pearson correlation coefficients, r, and statistical significance levels, p, are given in Table 3. All correlations between surfactant parameters and PaO_2_/FiO_2 _were significant (p < 0.05), with the exception of the total protein correlation. The highest correlation was observed for minimum surface tension, phospholipid-to-protein ratio, SP-C, SP-A and DPPC in LA (Figure 3, Table 3). With respect to the correlation between surfactant parameters and minimum surface tension, the highest correlation was found for DPPC in LA and SP-B in BALF (Table 3).

**Table 3:** Correlational analysis of ARDS data (T0 – T2)^§^

	**r**	**p**
(A)		

PaO_2_/FiO_2 _vs. γ min	-0.831	< 0.001
PaO_2_/FiO_2 _vs. phospholipid-to-protein ratio	0.662	< 0.001
PaO_2_/FiO_2 _vs. SP-C	0.644	< 0.001
PaO_2_/FiO_2 _vs. SP-A	0.627	< 0.001
PaO_2_/FiO_2 _vs. DPPC in LA	0.590	0.003
PaO_2_/FiO_2 _vs. phosphatidylglycerol	0.538	< 0.001
PaO_2_/FiO_2 _vs. neutrophils	-0.370	0.02
PaO_2_/FiO_2 _vs. SP-B	0.448	0.003
PaO_2_/FiO_2 _vs. total BALF protein	-0.238	0.12

(B)		

γ min vs. DPPC in LA	-0.754	0.012
γ min vs. SP-B	-0.708	0.002
γ min vs. total protein	0.641	0.007
γ min vs. SP-A	-0.598	0.02
γ min vs. neutrophils	0.553	0.05
γ min vs. phospholipid-to-protein ratio	-0.510	0.04
γ min vs. SP-C	0.281	0.29
γ min vs. phosphatidylglycerol	0.008	0.98

^§ ^Pearson correlation was performed between (A) surfactant compositional and functional parameters and PaO_2_/FiO_2 _and (B) between surfactant components and minimum surface tension (γ min) of the surfactant isolates for ARDS patients at T0, T1 and T2. The Pearson correlation coefficient, r, and the statistical significance, p, is given.

## Discussion

The aim of the current study was to investigate the time course of surfactant changes in patients with direct ARDS due to pneumonia or aspiration, a patient group that has recently been shown to be different from indirect ARDS patients with respect to imaging analysis, lung elasticity, recruitment capacity and frequency of additional organ failure, compared to ARDS patients with an extrapulmonary trigger event (indirect ARDS) [33,34,17]. As the frequency of additional organ failure has been linked to the outcome of ARDS patients [35], patients with direct ARDS may also have a slightly better prognosis. Considering these data, we focused on patients with direct ARDS. Serial bronchoalveolar lavages were performed at an early, intermediate and later stage of the disease and analyzed for lipid and protein composition and for surface properties of pulmonary surfactant. In accordance with previous reports [3-6], severe alterations of the pulmonary surfactant system were encountered in early direct ARDS, both, when being compared to the herein described group of healthy non-ventilated individuals or to a previously studied group of mechanically ventilated patients with cardiogenic lung edema reflecting a kind of ventilated control group in absence of significant inflammatory lung disease [6]. A considerable improvement in surfactant composition and function was noted over time, with some parameters reaching the normal range (such as SP-A, SP-B and large surfactant aggregate content), while others still remained different from controls (such as e.g. extent of phosphatidylcholine dipalmitoylation) at T2. Notably, the minimum surface tension of the isolated large surfactant aggregate fraction, although significantly improved over time, ranged at ~13 mN/m at T2, and thus was still highly elevated compared to healthy controls (~1 mN/m). Between T0 and T2, a highly significant correlation between gas exchange data and surfactant properties was encountered. The extent of surfactant improvement was significantly higher in survivors as compared to non-survivors.

To our best knowledge, only limited and conflicting data exist with regard to surfactant properties during the time course of ARDS. In an early study, Pison et al. [4] investigated pulmonary surfactant in a cohort of patients with ARDS after multiple trauma. In contrast to our study, these authors found a progressive deterioration of surfactant properties in the majority of parameters investigated. For example, total BALF protein remained unchanged during the first seven days of the disease in patients with high ARDS score, and the relative content of phosphatidylcholine declined significantly between day 0 and day 14. The reason for the discrepancy with our data is currently unclear, but differences in the triggering event (indirect in their study versus direct ARDS in our study) may play a role. Greene et al. [24] analyzed surfactant-associated proteins SP-A, SP-B and SP-D in patients at risk for ARDS, and during the time course of ARDS of unspecified etiology. In line with our data, they found decreased SP-A and SP-B levels compared to controls, but SP-A remained consistently low during the observation period (14 days). Another study [36] investigated serial changes in phospholipid composition in ARDS and found that phospholipid properties are partially improved during the time course in moderate and mild respiratory failure, but not in severe respiratory failure. Nakos et al. [37] investigated surfactant changes 0, 3 and 6 days after onset of ARDS. In accordance to our data, a decrease in lavagable proteins and improvements of the BALF cellular profile over time was visible. Additionally, PaO_2_/FiO_2 _values increased from 120 mm Hg (early ARDS) to 180 mm Hg (late ARDS). In contrast to our study, however, no improvement in the profile of phospholipid classes was found throughout the observation period, and total phospholipids decreased between 0 and 6 days.

In view of our data we suggest that dipalmitoylated phosphatidylcholine (DPPC), SP-B and SP-C are the probably most informative surfactant compounds that may explain the incomplete recovery of surface activity of the LA fraction after 7–9 days of ventilation. DPPC was reduced to less than 50% of control values at T0 and reached only ~65% of control values at T2. The reason for this profound and persistent reduction is currently unclear. Contamination with non-surfactant phospholipids, in our opinion, does not play a major role, because the analysis was performed with isolated large surfactant aggregates, which represent freshly secreted surfactant material. This assumption is further supported by the observation of a superimposable molecular species pattern of PC from LA being either prepared by means of sodium bromide gradient centrifugation according to Shelley et al. [38] or by means of high speed centrifugation at 48 000 × g (data not given in detail). We suggest that disturbances in surfactant phosphatidylcholine metabolism, for example, disturbances in the deacylation-reacylation pathway ("remodeling") [39,40] are largely responsible for this persistent depression of DPPC levels in BALF. However, further experiments are needed to clarify this issue. In addition, the hydrophobic surfactant proteins, which are known to dramatically enhance film stability under compression and adsorption facilities, were reduced to ~50% of controls, and only partially recovered during the later time course. It has also to be noted that our currently applied technique for measurement of SP-B and SP-C does not allow for the differentiation between intact mature SP-B/C and degradation products of these proteins, the generation of which may additionally exert a detrimental effect on surface activity of the LA fraction. Likewise, the underlying reason for the persistent suppression of the hydrophobic surfactant proteins despite full recovery of SP-A and unchanged SP-D values is currently unclear.

The study is limited in that only a few of the ARDS patients studied herein were mechanically ventilated in full accordance with the low-stretch strategy. Unfortunately, the vast majority of patients had already been recruited into this study before mechanical ventilation with low tidal volumes has been recognized as an important strategy to decrease mortality and before low-stretch ventilation has been applied routinely to ARDS patients. Therefore, we can not completely exclude that the time course of surfactant alterations may be different in ARDS patients treated with low tidal volumes.

An interesting aspect of the study is the observation that non-surviving patients displayed more unfavourable surfactant changes as compared to survivors who seemed to recover more quickly. This may suggest a causal association between surfactant function and outcome. However, it has to be taken into consideration that non-survivors were, at least at T0, ventilated with significantly higher lung volumes as compared to survivors. In line with the proposed relationship the herein described, more severe, surfactant changes in the non-survivor group may have induced the use of a more aggressive ventilatory approach. On the other hand it is well known, that ventilation with high tidal volumes may result in alterations of the pulmonary surfactant system [41]. Therefore, it is also imaginable that higher tidal volumes are the underlying mechanism for the observed more impaired surfactant function in the non-survivors and may in this way contribute to poorer outcome. Eventually, it is possible that higher tidal volumes in non-survivors may explain the differences in outcomes independent of surfactant function.

Do the data we present here help us to better understand the results of previous clinical trials, and to improve the design of future trials focusing on surfactant treatment in ARDS?

Aside from some smaller phase II studies employing natural surfactant preparations [18,19,22] three larger randomized, double-blind, placebo controlled, phase III trials using synthetic (Exosurf [42]) or recombinant SP-C based (Venticute [17]) surfactant preparations have yet been published. In the Exosurf trial, a fully synthetic phospholipid mixture containing tyloxapol was administered via continuous aerosolization to ARDS patients over a time period of 5 days. Neither gas exchange nor survival was different between placebo and verum groups [42] and this has been attributed to the overall much too low dose (~5 mg/kg body weight of phospholipids per day) being applied, and the high sensitivity of Exosurf towards inhibition. In contrast, a significant improvement in gas exchange was encountered in the two phase III studies assessing Venticute in ARDS patients [17] in the first 24 h after surfactant treatment, but not after 24 h. 46% of these patients had direct lung injury due to aspiration or pneumonia, and Venticute was administered up to four times at a dose of 50 mg/kg body weight phospholipids within the first 24 h. No additional treatment was performed after 24 h. Despite the beneficial effect on gas exchange, 28 d mortality was the same in the verum and the placebo group.

Considering the data that we present here, it seems reasonable to speculate that the duration of treatment in the Venticute trials was not long enough to ascertain an enduring effect of surfactant treatment on gas exchange, although there is only limited information available with regard to surfactant properties in response to surfactant treatment. As outlined in one recent investigation in surfactant treated ARDS patients, application of up to 500 mg/kg body weight of a calf lung surfactant extract in the first 24 h did not result in a persistent improvement of minimum surface tension values 72 h after the start of treatment (γ min value of ~15 mN/m) [21]. Even if the initial PaO_2_/FiO_2 _ratio has failed to show a predictive value in ARDS patients, it has been shown that missing improvement in pulmonary function during the first week indicates worse outcome. Thus, *persistent *improvement in oxygenation, along with the possibility to de-escalate the ventilatory regimen, may indeed promote better outcome in ARDS. In this line of reasoning, multiple surfactant dosing with persistent improvement in gas exchange may ultimately improve outcome in ARDS.

## Conclusion

We conclude that severe disturbances to surfactant composition and function occur early in direct ARDS due to pneumonia or aspiration, which only gradually resolve in the further time course of 8 days. These disturbances mostly affect the essential phospholipids dipalmitoylated phosphatidylcholine and phosphatidylglycerol and the surfactant-associated proteins SP-A, SP-B and SP-C. Correlational analysis suggests that the reduction of DPPC has the most significant association with the surfactant function impairment. These results may have impact on future strategies for surfactant therapy regarding the optimal composition and duration of surfactant administration.

## List of abbreviations

APACHE II scores on the acute physiology and chronic health evaluation

ARDS acute respiratory distress syndrome

BALF bronchoalveolar lavage fluid

CRP C-reactive protein

DPPC dipalmitoylphosphatidylcholine

FAME fatty acid methyl ester

HPTLC high performance thin-layer chromatography

HPLC high performance liquid chromatography

LA large surfactant aggregates

PaO_2_/FiO_2 _mean oxygen tension in arterial blood/inspiratory oxygen fraction

PC phosphatidylcholine

PEEP positive end-expiratory pressure

PG phosphatidylglycerol

PIP peak inspiratory pressure

PL phospholipid

PNEU severe pneumonia

PPQ phospholipid-to-protein ratio

SP-A, B, C, D surfactant proteins A, B, C, D

TLC thin-layer chromatography

## Competing interests

The author(s) declare that they have no competing interests.

## Authors' contributions

RS carried out the surfactant analyses and wrote the manuscript. PM helped coordinating the study. CR participated in the measurement of surfactant biophysics. MW performed the measurement of surfactant proteins, BAL fluid total protein content and cell differential. DW and TK carried out the bronchoalveolar lavages and helped acquiring the data. WS was involved in the design of the study and contributed to the writing of the manuscript with comments. AG conceived the study, participated in the design and helped drafting the manuscript. All authors read and approved the final manuscript.
